# Genomic Prediction for Whole Weight, Body Shape, Meat Yield, and Color Traits in the Portuguese Oyster *Crassostrea angulata*

**DOI:** 10.3389/fgene.2021.661276

**Published:** 2021-07-08

**Authors:** Sang V. Vu, Wayne Knibb, Cedric Gondro, Sankar Subramanian, Ngoc T. H. Nguyen, Mobashwer Alam, Michael Dove, Arthur R. Gilmour, In Van Vu, Salma Bhyan, Rick Tearle, Le Duy Khuong, Tuan Son Le, Wayne O’Connor

**Affiliations:** ^1^GeneCology Research Centre, University of the Sunshine Coast, Sippy Downs, QLD, Australia; ^2^School of Science, Technology and Engineering, University of the Sunshine Coast, Sippy Downs, QLD, Australia; ^3^Northern National Broodstock Center for Mariculture, Research Institute for Aquaculture Number 1, Hai Phong, Vietnam; ^4^Department of Animal Science, College of Agriculture and Natural Resources, Michigan State University, East Lansing, MI, United States; ^5^Queensland Alliance for Agriculture and Food Innovation, The University of Queensland, Saint Lucia, QLD, Australia; ^6^NSW Department of Primary Industries, Port Stephens Fisheries Institute, Taylors Beach, NSW, Australia; ^7^Consultant, Orange, NSW, Australia; ^8^School of Animal and Veterinary Science, The University of Adelaide, Adelaide, SA, Australia; ^9^Faculty of Environment, Ha Long University, Uong Bi, Vietnam; ^10^Research Institute for Marine Fisheries, Ngo Quyen, Hai Phong, Vietnam

**Keywords:** genetic improvement, genomic selection, high density SNP markers, prediction accuracy, genetic parameters

## Abstract

Genetic improvement for quality traits, especially color and meat yield, has been limited in aquaculture because the assessment of these traits requires that the animals be slaughtered first. Genotyping technologies do, however, provide an opportunity to improve the selection efficiency for these traits. The main purpose of this study is to assess the potential for using genomic information to improve meat yield (soft tissue weight and condition index), body shape (cup and fan ratios), color (shell and mantle), and whole weight traits at harvest in the Portuguese oyster, *Crassostrea angulata*. The study consisted of 647 oysters: 188 oysters from 57 full-sib families from the first generation and 459 oysters from 33 full-sib families from the second generation. The number per family ranged from two to eight oysters for the first and 12–15 oysters for the second generation. After quality control, a set of 13,048 markers were analyzed to estimate the genetic parameters (heritability and genetic correlation) and predictive accuracy of the genomic selection for these traits. The multi-locus mixed model analysis indicated high estimates of heritability for meat yield traits: 0.43 for soft tissue weight and 0.77 for condition index. The estimated genomic heritabilities were 0.45 for whole weight, 0.24 for cup ratio, and 0.33 for fan ratio and ranged from 0.14 to 0.54 for color traits. The genetic correlations among whole weight, meat yield, and body shape traits were favorably positive, suggesting that the selection for whole weight would have beneficial effects on meat yield and body shape traits. Of paramount importance is the fact that the genomic prediction showed moderate to high accuracy for the traits studied (0.38–0.92). Therefore, there are good prospects to improve whole weight, meat yield, body shape, and color traits using genomic information. A multi-trait selection program using the genomic information can boost the genetic gain and minimize inbreeding in the long-term for this population.

## Introduction

The Portuguese oyster, *Crassostrea angulata*, is becoming an important aquaculture mollusk species in various parts of the world, including Asia and Europe (Grade et al., 2016; Vu et al., 2017a; Gagnaire et al., 2018). The hatchery and production technologies are well established for this species. A genetic improvement program was established in Vietnam in 2014 using stock from three hatcheries to form the base population (Vu et al., 2017b). Initially, the breeding program focused solely on increasing the whole weight at harvest using traditional family selection approaches. The genetic evaluation of the first three generations (generation interval is 1 year) of the program showed a considerable genetic gain of around 6.0% per generation in the whole weight at harvest (Vu et al., 2019). Other traits such as meat yield, body shape (“cup” and “fan” ratios), and color of the shell and mantle have more recently been evaluated and incorporated into the breeding program due to an increasing market demand for these traits (Vu et al., 2019, 2020). Seafood color, especially for oysters that are usually sold live or in the half-shell, affects consumers’ preferences and their willingness to buy because certain colors are perceived to be associated with better flavor and quality (Kahn and Wansink, 2004; Xing et al., 2018). In addition, shell shape has been reported to be related to meat yield in mollusk species (Gimin et al., 2004). Misshapen animals fetch a lower price than well-formed oysters (Lee et al., 2004). Previous studies showed that the heritability for meat yield, body shape, and color traits varied from 0.13 to 0.57 and has potential for genetic improvement (Vu et al., 2019, 2020). Conventional selection, however, is less efficient for traits that cannot be measured in living animals (Meuwissen et al., 2013). This is the case for meat yield and mantle color as these traits require slaughter before measurements can be taken. The current breeding program relies on measurements of meat yield and mantle color from the siblings of the selection candidates and on correlated traits. As a result, selection accuracy has been low and genetic progress has been slow for these traits. An alternative would be to add genomic selection for these difficult-to-measure traits to the breeding program; it is considered a more efficient way to speed up the rate of genetic progress and increase the prediction accuracy (Muranty et al., 2014).

Genomic selection is now widely used in many agricultural species, and it can significantly improve traits that are determined by many loci with small effects (Robledo et al., 2018b). Genomic selection can be used to estimate the breeding values of individuals with no pedigree information and can more accurately predict breeding values than traditional selection based on pedigree records because it uses genetic markers to build a genomic relationship matrix to be used to estimate the breeding values for individuals that better approximate *realized* relationships (Goddard and Hayes, 2007). The advantages of genomic selection over traditional pedigree-based approaches in terms of the accuracy of the predictions for polygenic traits have been well established for aquaculture species (Vallejo et al., 2017; Gutierrez et al., 2018, 2019; Hollenbeck and Johnston, 2018; Joshi et al., 2019). In addition, genomic selection is extremely useful for exploring within-family variability to increase the rates of genetic gain and to reduce generation intervals (Georges et al., 2019). In aquaculture species, the accuracy of genomic prediction reported for growth, carcass, and meat quality traits as well as disease resistance traits ranged between 0.16 and 0.83 (Castillo-Juárez et al., 2015; Zenger et al., 2018; Joshi et al., 2019; Liu et al., 2019; Yoshida et al., 2019). Genomic prediction has not yet been widely adopted for mollusk breeding, but there are a few examples of its use for traits such as growth and disease resistance in Pacific oysters, *Crassostrea gigas* (Gutierrez et al., 2018, 2019), and pearl quality in pearl oysters, *Pinctada maxima* (Jones et al., 2017). To our knowledge, there are no reports on the genomic prediction for whole weight in Portuguese oysters nor for meat yield (soft tissue weight and condition index), body shape, and color (shell and mantle) in any other oyster species.

The purpose of this study was to (i) estimate the genomic heritability and genetic correlations for meat yield, color traits, harvest whole weight, and body shape and (ii) evaluate the accuracy of genomic prediction for these traits in Portuguese oysters.

## Materials and Methods

### Oyster Samples

The Portuguese oysters used in this study originated from a breeding program to improve the whole weight at harvest in Vietnam (Vu et al., 2019). The oysters were produced in a hatchery and then raised in the open ocean from spat (2–4 mm) until harvest. The oyster families were separately raised in the ocean. Tissue samples were collected from the oysters at harvest after about a 9-month period of culture. Information on the animals included sire, dam, spawning date, harvest date, and rearing condition. Phenotypic measurements taken on the oysters included whole weight, body shape, meat yield, and color traits. Tissue samples were collected from a total of 647 oysters: 188 oysters representing 57 full-sib families from the first generation and 459 oysters representing 33 full-sib families from the second generation. The number per family ranged from two to eight oysters for the first and 12–15 oysters for the second generation. Most of the oysters from the second generation were progeny of the first generation of oysters (Vu et al., 2021b). All tissue samples were preserved in 80% ethanol, stored at −80°C, and sent to Diversity Array Technology Pty. Ltd., Canberra, Australia, for sequencing.

### Traits Studied

#### Growth

The oyster shells were cleaned in water before taking the measurements. The whole weight of oysters at harvest was recorded using electronic scales with an accuracy of 0.01 g.

#### Body Shape

The cup and fan ratios were used as indicators of the body shape of the oysters. The cup ratio was calculated by dividing the shell width by the shell depth, and the fan ratio was calculated by dividing the shell length by the shell depth (Walton et al., 2013).

#### Meat Yield

Electronic scales with an accuracy of 0.01 g were used to measure the soft tissue weight. The condition index was calculated as soft tissue weight multiplied by 100/(whole weight minus shell weight) (Lawrence and Scott, 1982).

#### Color

The shell and mantle colors of oysters at harvest were measured using a FRU colorimeter WR10 8 mm model (Giang et al., 2019) *via* a CIELab L^∗^a^∗^b colorimeter which determines the color as a number under a device-independent 3D color model (Leon et al., 2006). The three dimensions in the model are L^∗^ (whiteness), a^∗^ (redness), and b^∗^ (yellowness). The values of L^∗^ range from 0 for black to 100 for white; a^∗^ values are negative for green and positive for red; and b^∗^ values are negative for blue and positive for yellow (b^∗^ = zero, neutral color) (Giang et al., 2019).

### DNA Extraction, Library Construction, and Genotyping

The frozen oyster mantle samples were sequenced using DArTSeq^TM^ (Diversity Arrays Technology Pty. Ltd., Canberra, Australia) at a high marker density of 2.5 million sequences per sample. Genomic DNA was extracted and purified by Diversity Array Technology Pty. Ltd. (Supplementary Table S1). The SNP development and analysis were as described by Kilian et al. (2012). Briefly, DNA samples were digested with *Pst*I–*Sph*I (Kilian et al., 2012; Vu et al., 2021a) and then ligated using two different adaptors with two different restriction enzyme overhangs. The *Pst*I-compatible adapter consists of Illumina’s flow-cell attachment, a sequencing primer, and a “staggered” varying-length barcode region (Elshire et al., 2011). The reverse adapter included a flow-cell attachment region and a *Sph*I-compatible overhang sequence. The PCR reaction used to amplify the mixed fragments of *Pst*I–*Sph*I following the reaction conditions comprised of an initial denaturation for 1 min at 94°C, followed by 30 cycles of 94°C for 20 s, 58°C for 30 s, and 72°C for 45 s, and a final extension at 72°C for 7 min.

The PCR products from each sample were fed to the c-Bot (Illumina) bridge PCR, followed by sequencing 77 nucleotide single-end reads on an Illumina Hiseq 2500. Reads with inaccurate barcode sequences were filtered out. Approximately 2,500,000 sequences per barcode/sample were used in marker calling. Finally, identical sequences were collapsed into “fastqcoll files,” which were “groomed” to correct for low-quality bases. The “groomed” fastqcoll files were used in a secondary pipeline incorporating DArT SNP and SilicoDArT (presence/absence of restriction fragments in representation), called analysis algorithm DArTsoft14, which clustered all tags from all libraries at a threshold of 3 for SNP calling. Technical parameters, especially the balance of read counts for the allelic pairs, were used to parse into separate SNP loci. Additional selection criteria included analysis of approximately 1,000 controlled cross-populations and testing for Mendelian distribution of alleles to assign consistency scores to classify high-quality/low-error rate markers. Calling quality was assured by a high average read depth per locus (the average across all markers was over 30 reads/locus).

The sample and marker statistics are given in Supplementary Tables S2, S3, respectively. There were 18,849 SNP markers identified from genotyping by sequencing. Quality control was conducted with a call rate of 50% of SNP presence in the samples, leaving 13,048 SNP markers retained for analysis. The missing genotypes were imputed using the SVS Suite software with a default setting, changing them to average values (Bozeman, 2010).

### Estimation of Narrow Sense Heritability and Genetic Correlations

Univariate linear mixed models were used to estimate the narrow sense heritabilities of the traits. The fixed effects fitted in the model were generation (age is a linear covariate nested within generation), sex determined at harvest (age based on spawning and harvest dates), and their interactions. The additive genetic effect and the residuals were used as random factors.

Heritability was calculated as the ratio of $\sigma_{a}^{2}$ on $\sigma_{p}^{2}$, where $\sigma_{a}^{2}$ is the additive genetic variance and $\sigma_{p}^{2}$ is the phenotypic variance calculated as $\sigma_{p}^{2}=\sigma_{a}^{2}+\sigma_{e}^{2}$ and $\sigma_{e}^{2}$ is the residual variance.

Genetic correlations between traits were estimated using a bivariate version of the same model (Mrode, 2014). All analyses were conducted using the average information restricted maximum likelihood procedures in the SVS Suite software (Bozeman, 2010).

### Genomic Prediction

The accuracy of genomic prediction was estimated for all traits by fivefold cross-validation analysis (training set—80% and validation set—20%) with five replicates for each trait using gBLUP through the SVS Suite software (Bozeman, 2010). The gBLUP method assumes equal distribution and variance for individual SNP effects. The genome-wide complex trait analysis method was used to calculate the normalized genomic relationship matrix in our analysis (Yang et al., 2011). The gBLUP model is written in matrix notation as follows:

y=m+X⁢ß+M⁢a+e

where *y* is the vector of phenotypic observations (whole weight, body shape, meat yield, and color traits), *m* = overall mean, and *X* is the incidence matrix consisting of fixed effects (generation, sex, age, and interactions) in ß. The matrix M is the incidence matrix of genetic effects, and the genetic values are *g=Ma*, such that $g_{n\times 1}∼N\,\left(0\,n,G\,\sigma_{g}^{2}\right)$ and the genomic relationship matrix is $G_{n\times n}=\frac{M\,M^{\prime }}{\sum_{j=1}^{m}2\,p_{j}\,q_{j}}$ (VanRaden, 2008), in which $p_{j}^{\prime }\,s$ are the minor allele frequencies of the SNP genotypes (*q*_*j*_ = 1−*p*_*j*_) and $\sigma_{g}^{2}=\sum_{j=1}^{m}2\,p_{j}\,q_{j}\,\sigma_{a}^{2}$. Using the genomic best linear unbiased prediction (GBLUP) (VanRaden, 2008), genetic values were then fitted (on discovery population) and predicted (on validation population) by solving the mixed model equation.

The allele substitution effects (ASE) and fixed effect coefficients obtained from iterations and *k*-folds of the cross-validation analysis that gave the largest *R*^2^-value were used to predict the phenotypes of individual animals with the following model:

$$\hat{y}=X\,\hat{ß}+M\,\hat{\alpha }$$

where *$\hat{y}$* is the predicted phenotypes, *X* is the fixed effects matrix, $\hat{ß}$ is the fixed effect coefficient, M is the genotype matrix, and $\hat{\alpha }$ is the ASE values.

The fivefold cross-validation using the gBLUP method was conducted using the SVS Suite (Bozeman, 2010). The actual and predicted phenotypes were compared using linear regression analysis. The coefficient of determination (*R*^2^) from the regression analysis was used to assess the predictive ability of the gBLUP model for the traits studied. Furthermore, the animal effects were also predicted by traditional BLUP using the pedigree-based numerator relationship matrix for comparison with the genomic-based predictions described above. The pedigree summary of the oyster population in this study is given in Supplementary Table S4.

### Population Genetics

Nucleotide diversity was estimated using the method developed by Nei and Li (1979). Using an in-house computer program, the genotype file obtained from DArTSeq was converted to the variable call format (VCF). This file was used as an input for the online software *VCF2PopTree* (Subramanian et al., 2019) to obtain the pairwise divergence matrix of all combinations of oyster individuals. This matrix was then used to construct a neighbor-joining tree using the software MEGA (Kumar et al., 2018). Fixation index (*F*_*ST*_) was calculated using the nucleotide diversities of the total and sub-populations (Nei, 1973).

## Results

### Phenotypic Data

The summary statistics of the traits are shown in Table 1. The average whole weight at harvest calculated from the 647 oysters in this study was 51.07 g, with the data range within three standard deviations of the mean (Table 1). There was considerable variation in whole weight, with a wide range from 15.71 to 69.90 g. The shell and mantle color had a significant yellow component (mean b^∗^ significantly greater than zero) and was dark (L around 25% but with considerable variation). The average cup and fan ratios indicated a typical depth/width/length ratio of 1:1.6:2.9. The largest variation levels were also found in color traits of tissues and shells, especially for shell color a^∗^, with values from −2.07 to 54.96, and mantle color L^∗^, from 7.24 to 63.70.

**TABLE 1:** Summary of the phenotypic data.

Groups	Traits	Unit	*n*	Mean	SD	Min	Max
Growth	Whole weight	g	647	51.07	16.13	15.71	69.90
Body shape	Cup ratio	Ratio	361	1.61	0.33	0.74	2.60
	Fan ratio	Ratio	361	2.93	0.72	1.46	5.07
Meat yield	Soft tissue weight	g	490	9.55	3.13	1.05	19.92
	Condition index	Index	284	67.78	21.65	14.29	99.93
Color	Shell color a*	CIELab	306	2.35	3.71	−2.07	54.96
	Shell color b*	CIELab	306	11.58	4.25	3.99	43.02
	Shell color L*	CIELab	306	23.63	12.30	6.49	69.71
	Mantle color a*	CIELab	448	4.40	3.46	0.16	24.60
	Mantle color b*	CIELab	448	10.11	4.01	1.02	23.30
	Mantle color L*	CIELab	448	24.21	8.73	7.24	63.70

### Heritability and Genetic Correlation

The genomic heritability (*h*^2^), the proportion of total variance explained by the SNP markers, ranged from 0.14 to 0.77 (Table 2). The heritability of the shell color traits ranged from moderate to high (0.14–0.54), and the estimates of heritability were 0.13 for a^∗^ (red), 0.26 for b^∗^ (yellow), and 0.34 for L^∗^ (lightness). For the mantle, these traits had heritabilities of 0.54 for red, 0.49 for yellow, and 0.34 for lightness. The heritability estimates for meat yield traits were relatively high (0.43–0.77). Similarly, the estimate of heritability for whole weight was quite high at 0.45. However, the heritability estimates for body shape using the indicators of cup and fan ratios were low to moderate at 0.24 and 0.33, respectively.

**TABLE 2:** SNP heritability (*h*^2^) and genetic correlations (*r*_*g*_) of whole weight with other traits.

Groups	Traits		Heritability			Genetic correlation	
				*h*^2^	SE	*r*_*g*_	SE
Growth		Whole weight		0.45	0.06		
Body shape		Cup ratio		0.24	0.10	0.14	0.24
		Fan ratio		0.33	0.08	0.08	0.15
Meat yield		Soft tissue weight		0.43	0.07	0.63	0.10
		Condition index		0.77	0.07	0.18	0.15
Color		Shell color a*		0.14	0.07	–0.13	0.22
		Shell color b*		0.26	0.09	–0.02	0.13
		Shell color L*		0.34	0.09	0.34	0.19
		Mantle color a*		0.54	0.06	–0.13	0.13
		Mantle color b*		0.49	0.07	–0.14	0.15
		Mantle color L*		0.34	0.09	0.14	0.19

### Genetic Correlations

The positive genetic correlation between whole weight and soft tissue weight was high at 0.63, suggesting that selection for improved whole weight can result in desirable changes in soft tissue weight. However, low or insignificant genetic correlations were observed between whole weight and the other traits: color traits (−0.14 to 0.34), condition index (0.18), and body shape (0.08–0.14), implying that the selection for growth would result in little or no changes in these traits in this population. In addition, the genetic correlations between whole weight and all color traits were not only small, but they all had large standard errors and were therefore not significant. The genomic genetic correlations (*r*_*g*_) of whole weight with the other traits are presented in the Table 2.

### Accuracy of Genomic Prediction

Table 3 shows the prediction accuracy of genomic selection for the traits using the fivefold cross-validation method. High prediction accuracies using GBLUP were found for meat yield and color traits, where measurements cannot be taken on living animals. The accuracies were 0.71 for soft tissue weight and 0.49 for condition index. For the color traits, the accuracies ranged between 0.38 and 0.92. The GBLUP prediction accuracy was comparatively high for the harvest whole weight at 0.74; similarly, the body shape traits also showed a high accuracy, with 0.40 for cup ratio and 0.63 for fan ratio. Across all traits, the GBLUP predictions had higher prediction accuracies than the traditional BLUP prediction accuracies.

**TABLE 3:** Genomic prediction accuracy of the traits with GBLUP and traditional BLUP.

Factors		Single variate linear mixed model	

Groups	Traits	GBLUP	BLUP
Growth	Whole weight	0.74	0.66
Body shape	Cup ratio	0.40	0.29
	Fan ratio	0.63	0.55
Meat yield	Soft tissue weight	0.71	0.51
	Condition index	0.49	0.22
Color	Shell color a*	0.92	0.46
	Shell color b*	0.62	0.65
	Shell color L*	0.83	0.66
	Mantle color a*	0.87	0.67
	Mantle color b*	0.89	0.44
	Mantle color L*	0.38	0.14

**FIGURE 1:**
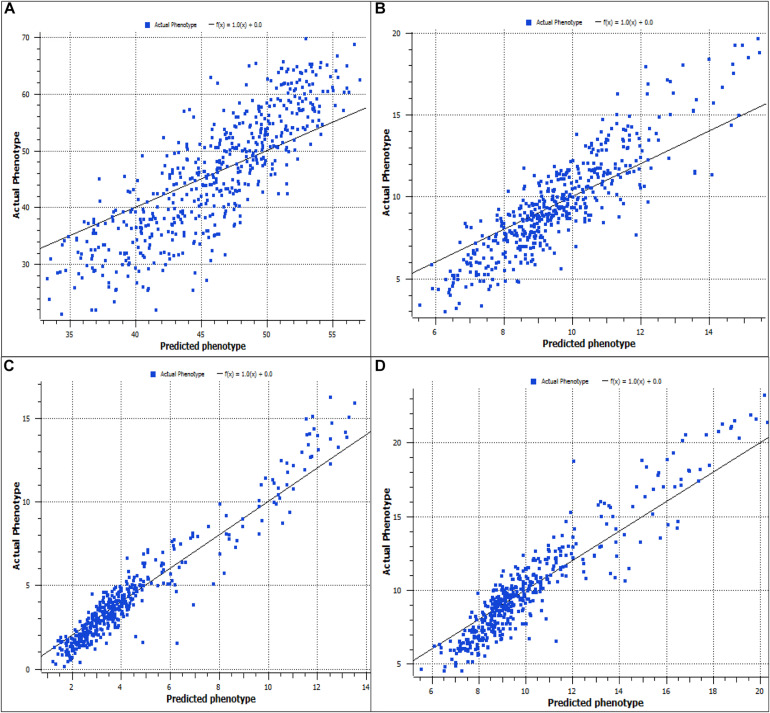
Prediction accuracy for whole weight **(A)**, soft tissue weight **(B)**, mantle color a **(C)**, and mantle color b **(D)**.

The association test of predicted and actual phenotypes for four predominant traits are presented in Figure 1A for the whole weight, Figure 1B for soft tissue, Figure 1C for mantle color a^∗^, and Figure 1D for mantle color b^∗^. The *R*^2^ values varied from 0.37 for the mantle color L^∗^ to 0.91 for the shell color a^∗^.

## Discussion

Our results have made five important discoveries that can be used to improve the genetic progress of the current breeding program for Portuguese oysters. Specifically, they answer the following questions.

### Is There Any Genetic Variation in the Traits Studied?

Our analysis indicated a large genetic variation, with estimates of genomic heritability falling in the range of 0.14–0.77. Our results were somewhat higher than those we have previously obtained with the traditional pedigree-based relationship matrix in the same population (Vu et al., 2019). The genomic heritability for meat yield and body shape, in particular, was much larger than that reported using a pedigree-based estimation (Vu et al., 2019). Similarly, the estimates of heritability based on SNP markers for mantle color traits (*h*^2^ = 0.34–0.54) were around twice those based on pedigree (*h*^2^ = 0.15–0.33) (Vu et al., 2020). These differences between genomic and pedigree heritability estimates of meat yield, body shape, and color traits are most likely due to errors in pedigree recording between spawning and harvesting. These results suggest that genomic selection can be extremely efficient for traits that cannot be measured directly on the selection candidates especially since oyster breeding involves a few large families, and pedigree information is difficult to record accurately. Unfortunately, there have been no other studies using a genomic selection approach to estimate heritability for meat yield, color, and body shape traits in mollusk species that could be used for comparison. Finally, the estimation of heritability for whole weight in this study was significantly higher than that reported in the Pacific oyster by Gutierrez et al. (2018) (*h*^2^ = 0.45 vs. 0.35, respectively). However, estimated heritability in genomic selection can also depend on species, sample size, marker density, relationship of reference and validation population, and statistical methods for analysis (Georges et al., 2019). It should be noted that our population is highly inter-related, and the high estimates probably only pertain to selection within that population.

### Does This Genomic Analysis Predict That Selection for Harvest Whole Weight Will Affect Body Shape, Meat Yield, and Color?

The genetic correlations obtained with the GBLUP analysis were generally similar to those obtained from the traditional BLUP method (Vu et al., 2019). The high and positive genetic correlation between harvest whole and soft tissue weight indicates that these two traits are controlled by some common sets of genes. This result is in line with the previous estimate by Vu et al. (2019) using the BLUP method (0.63 for GBLUP vs. 0.50 for BLUP). Therefore, soft tissue weight will increase along with the harvest whole weight under the selection for harvest whole weight. Meanwhile, the small positive genetic correlations found between harvest whole weight and body shape show that there is some/limited potential to improve these traits by selection for harvest whole weight, and any genetic progress will be very slow. Therefore, trait groups such as whole weight, body shape, and meat yield can improve in the same direction, and this suggests that the selection for one trait (harvest whole weight) will lead to an improvement in these other traits. In contrast, the negative and positive but low genetic correlations between whole weight and color traits found in this study agree with those reported in the same population using the pedigree BLUP method (Vu et al., 2020). Consequently, our results suggest that no potential exists to improve color traits by selection for harvest whole weight. Taken together, this study provides fundamental information to better understand the genetic architecture of quantitative complex traits in Portuguese oysters.

### Is Genomic Selection Reliable for the Traits Studied?

Our study reports, for the first time, the predictive accuracy of genomic selection for color and meat yield traits in a mollusk species. The predictive accuracies using the GBLUP model for meat yield and color traits were significantly higher than those obtained from the pedigree BLUP model, suggesting that the genomic selection for these traits is more efficient than the pedigree-based selection. The predictive accuracies using the GBLUP method for meat yield and color traits are in good agreement with those reported in banana shrimp (Nguyen et al., 2019). However, these accuracies were higher than those obtained for growth-related traits in the Pacific oyster (Gutierrez et al., 2018), Pacific white leg shrimp (Wang et al., 2017), and yellow drum (Liu et al., 2019). In addition, the accuracies from our study are high due to the close relationships of the sampled oysters. The differences among the studies could originate from the SNP marker density, the set of SNP markers used for the analysis (Liu et al., 2019), the training population size (Andonov et al., 2017; Zenger et al., 2018; Georges et al., 2019), the relationship between the training and validation datasets (Hayes and Goddard, 2001), the relationships between individuals in the reference population (Hayes et al., 2009), and the heritability of the traits (Daetwyler et al., 2008; Georges et al., 2019). To the best of our knowledge, no studies have reported the prediction accuracy of genomic selection for body shape in any other aquaculture species. Collectively, across aquaculture species, our estimates of accuracies for whole weight, body shape, meat yield, and color traits in Portuguese oysters fall in the range observed in other aquaculture species (Zenger et al., 2018). The high levels of prediction accuracy open new selection opportunities to improve meat yield and color traits in the Portuguese oyster.

### Does Population Stratification Affect the Results of This Study?

**FIGURE 2:**
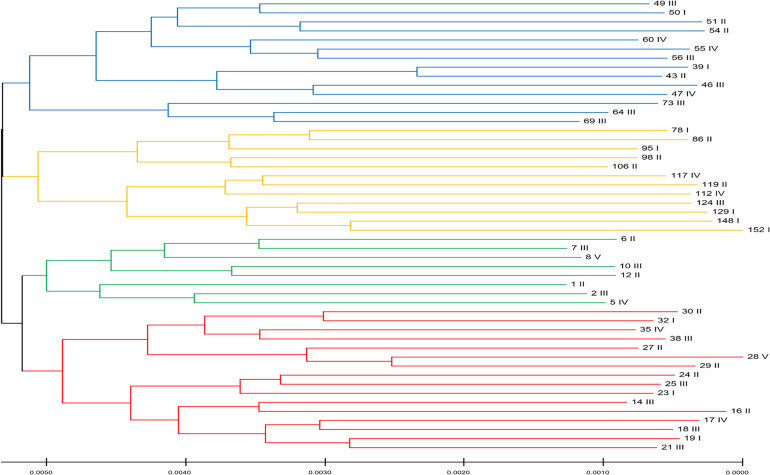
The structure of the population for this Portuguese oyster population in the first generation. Colors represent four distinct clusters based on genetic similarities. Roman numerals represent the whole weight category that the individual belong to: I: 30–40, II: 40–50, III: 50–60, IV: 60–70, and V: >70 g. This figure demonstrates that the oysters in the population do not cluster based on their whole-body weight but only based on their genetic relationship.

To quantify the genetic variation in the data, we estimated the nucleotide diversity, which produced a value of 0.009, suggesting 0.9% variation in the population. This estimate was slightly less than that observed for the Pacific oyster (1.2%) (Zhang et al., 2012). One of the confounding factors in genomic selection method is the structure of the populations, owing to the effects of genetic drift (Goddard and Hayes, 2007). Some of the genetic markers can, by chance alone, correlate with the traits due to the familial or pedigree structure of the populations compared. To examine the genetic relatedness among the first-generation animals, we selected 50 individuals, excluding their siblings. We categorized these oysters based on their whole-body weight as I: 30–40, II: 40–50, III: 50–60, IV: 60–70, and V: >70 g. We then constructed a neighbor-joining tree (Figure 2), which revealed four distinct clusters (four colors) in the population based on their genetic similarities. The fixation index (*F*_*ST*_) of 0.13 among these clusters suggested the existence of a population structure. However, it is evident from Figure 2, which shows that the whole weight of the members of each cluster varies significantly within a cluster. Therefore, these results suggest that the population structure observed in the data does not correlate with the phenotypic trait (whole weight) evaluated in this study. Hence, population stratification is unlikely to influence the results of this study.

### Are There Any Prospects for Using Genomic Selection in This Portuguese Oyster Population?

Genomic selection programs can be improved by using updated estimates of genetic parameters and benefiting from the higher predictive accuracy for quantitative complex traits, especially meat yield and color traits, in the Portuguese oyster. The favorably positive genetic correlations between whole weight, body shape, and meat yield traits found in this study suggest that there is little need to use a selection index to improve these related traits. To further illustrate, the heritability and genetic correlations from our study were used in SelAction (Rutten et al., 2002) to simulate the selection process and indicated that the traits whole weight, meat yield, and body shape can be improved simultaneously. The selection for enhancing whole weight results in beneficial changes in the other traits. However, it is necessary to use a selection index to improve color traits as well as weight traits due to the low genetic correlations between whole weight and color traits.

Collectively, the results from this study showed that there are prospects for the application of genomic selection to improve a range of complex traits in the Portuguese oyster. Consequently, further studies should be carried out to collect further information on predictive power and potential genetic gain. Firstly, the training population size needs to be increased to obtain a more accurate prediction (Goddard, 2009), i.e., we need to increase our sample size from the rather small population of 647 oysters used in this study to a much larger reference population which represents more families and generations. One strategy can be a mixture of low- and high-density panels, where the broodstock is sequenced at a higher marker density and the offspring are sequenced at a lower density, and then the lower density panels are imputed up to the higher density for the genomic prediction (Yoshida et al., 2018; Tsairidou et al., 2020). This strategy allows a larger number of samples to be sequenced, thus a larger training population size. This has been done at medium density in Pacific oysters for growth-related traits or in salmon (Gutierrez et al., 2018). Secondly, the use of a genome-wide association study—informative SNPs instead of random SNPs—was able to assist in improving the predictive accuracy (Liu et al., 2019). Therefore, the use of a smaller number of informative SNPs may help to reduce the cost of genotyping in this population. Thirdly, the prediction equations were evaluated in the reference population in which both phenotypes and genotypes were available; validation should be carried out in a breeding population where the selection candidates are the training population, and validation is on subsequent generations. Fourthly, individual grouping methods such as mean weight of family based on genomic information should be considered in Portuguese oysters to improve the accuracy for further studies. These individual grouping methods have been shown to increase the predictive accuracy and resulted in a higher genetic gain (Chu et al., 2019).

From the discussion above, the optimization of genomic selection will reduce the generation interval and increase the genetic gain in this population. Lowering the generation interval may result in an increase in inbreeding rate in the long-term. Therefore, to balance between inbreeding rate and genetic gain, genetic improvement programs have applied optimal genomic selection methodology and strategies (Nielsen et al., 2011; Gorjanc and Hickey, 2018; Robledo et al., 2018a). For the Portuguese oyster sector, if genomic selection is integrated into the breeding program, it could reduce the generation interval below 1 year per generation. The lower generation interval contributes to a faster life cycle, resulting in a higher economic revenue due to saving labor and time. In addition, estimates of breeding values using genomic information are more accurate than those using physical tags in the pedigree, where errors may occur during spawning, larval rearing, or pedigree management. Therefore, genomic selection will allow a more accurate selection of oysters to become parents in the next generations. However, a major impediment to widely using genomic selection in the aquaculture industry is the cost of genotyping and phenotyping the selection candidates. The former can be dealt with by using low-density SNP panels that can balance between prediction accuracy and sequencing costs to achieve the accuracy needed for genomic selection (Lillehammer et al., 2013; Gutierrez et al., 2018; Tsairidou et al., 2020; Boudry et al., 2021; Vu et al., 2021a).

## Conclusion

The predictive accuracy using genomic selection was relatively high for all the traits studied. In addition, the estimates of heritability in the meat yield traits such as soft tissue weight and condition index were high, while those of color traits such as shell and mantle color were from low to high. The genomic selection for improvement of whole weight leads to desirable changes in other traits, such as meat yield and body shape traits, but will not affect color traits. Future breeding programs should combine all traits into a selection index to bring about higher revenues for aquaculture farmers and entrepreneurs.

## Data Availability Statement

The DNA sequence data used in this study is available and can be accessed through the USC Research Bank under doi 10.25907/00038. The phenotypic data is available by request from the corresponding authors.

## Ethics Statement

There are no requirements on ethics approval for mollusk species in Vietnam.

## Author Contributions

SV, WK, and WO’C conceived and designed the experiments and selected the samples for sequencing. SV and NN prepared, drafted, and edited the manuscript. SV, CG, MA, and SB analyzed the data. SS involved in the population structure analysis. TL and LK prepared the oyster tissue samples. SS, CG, MA, MD, AG, RT, IV, and WO’C participated in editing the manuscript. All authors read and approved the manuscript.

## Conflict of Interest

The authors declare that the research was conducted in the absence of any commercial or financial relationships that could be construed as a potential conflict of interest.
