# Supplementary material for: Genomic Prediction for Whole Weight, Body Shape, Meat Yield, and Color Traits in the Portuguese Oyster Crassostrea angulata
Source: Front Genet. 2021 Jul 8;12:661276. doi: 10.3389/fgene.2021.661276 (PMC8298027; doi:10.3389/fgene.2021.661276)
Supplement: Supplementary file 1 [file Data_Sheet_1.docx]

**Supplementary**

**Supplementary Table S1**. DNA extraction procedure using Dart-seq technology was as follows:

| **Procedure** | **Content** |
| --- | --- |
| Step 1 | Centrifuge plate at 2000 rpm for 3 minutes. |
| Step 2 | Carefully unseal the columns of PCR plate. |
| Step 3 | Pipette out all Ethanol using a multichannel pipette and place the plate in the incubator at 60°C to allow all ethanol to evaporate. |
| Step 4 | Lyse samples: For sample use 50µL of T1 Buffer and 6.25µL of proteinase K. Dispense 56.25µL of lysis solution into each sample, place the strip caps firmly and vigorously vortex the plate (use flat strip of 8 caps from Sarstedt to avoid leakage). Centrifuge plates briefly (30 to 60 sec at 1000 rpm) to collect any sample at the bottom of the wells. The tissue samples must be submerged in the solution. Incubate overnight at 60°C and to prevent any evaporation. |
| Step 5 | Clear Lysate: Centrifuge for 10 min at 3000 rpm (program 5 on the centrifuge). Aspirate as much clear lysate as possible without touching any tissue leftover, transfer into the labelled deep well plate. |
| Step 6 | Bind DNA to NucleoMag B-beads. Each sample need 6µL beads suspended in 90µL MB2. Measure Beads/MB2 volumes accurately and combine in a tube (15/50mL falcon tube/ 2mL Eppendorf tube for few samples), place cap on and mix well to suspend beads. Pour premixed Beads/MB2 solution into a reservoir, dispense 96µL to each sample. Ensure to gently agitate the reservoir at all times whilst dispensing to prevent the beads from settling down. Transfer the deep well plate to T100 robot. |
| Step 7 | Final extraction steps (washing and elution into 30 uls of Elution Buffer) is done on Tecan 100 robot using 96 tips head and DArT PL script. |

**Supplementary Table S2**. Basic statistics about the sequence data.

| **SNP statistics** | | Mean | Range |  |
| --- | --- | --- | --- | --- |
| Total SNPs | | 13,048 |  |  |
| AvgCountSnp | | 23.7 | 2.5 – 295.4 |  |
| AvgCountRef | | 33.6 | 2.5 – 451.5 |  |
| FreqHomSnp (%samples which score homozygote) | | 0.22 | 0 – 1 |  |
| FreqHets (%samples which score heterozygote) | | 0.11 | 0 – 0.68 |  |
| PIC SNP | | 0.28 | 0 – 0.5 |  |
| Call rate | The proportion of samples for which the genotype call is either '1' or '0', rather than '-'. | | | |

**Supplementary Table S3**. Descriptive population genetic estimates and statistics for SNPs

| Markers statistics | Mean | Minimum | Maximum |
| --- | --- | --- | --- |
| Call Rate | 0.74 | 0.20 | 1.0 |
| Minor Allele Frequency | 0.19 | 0.00077 | 0.5 |
| Major Allele Frequency | 0.81 | 0.50 | 0.999 |
| Expected HWE P | 0.5 | 0 | 1 |
| Minor Allele Count | 181.4 | 1 | 641 |
| Major Allele Count | 809.6 | 142 | 1294 |
| Missing Allele Count | 331.3 | 0 | 1034 |

**Supplementary Table S4**. The pedigree summary of the oyster population in this study

| \| **Sample** \| **Sire** \| **Dam** \| **Generation** \| \| --- \| --- \| --- \| --- \| \| 1 \| 1002791 \| 1000717 \| 1 \| \| 2 \| 1002262 \| 1001400 \| 1 \| \| 3 \| 1002262 \| 1001400 \| 1 \| \| 4 \| 1000185 \| 1002835 \| 1 \| \| 5 \| 1001051 \| 1001837 \| 1 \| \| 6 \| 1001462 \| 1000242 \| 1 \| \| 7 \| 1001927 \| 1001486 \| 1 \| \| 8 \| 1002466 \| 1000904 \| 1 \| \| 9 \| 1001462 \| 1000242 \| 1 \| \| 10 \| 1001896 \| 1001261 \| 1 \| \| 11 \| 1001875 \| 1002567 \| 1 \| \| 12 \| 1000541 \| 1002467 \| 1 \| \| 13 \| 1001875 \| 1002567 \| 1 \| \| 14 \| 1001358 \| 1000806 \| 1 \| \| 15 \| 1000574 \| 1001195 \| 1 \| \| 17 \| 1000185 \| 1000551 \| 1 \| \| 18 \| 1001171 \| 1001446 \| 1 \| \| 19 \| 1000961 \| 1001624 \| 1 \| \| 20 \| 1000574 \| 1001195 \| 1 \| \| 21 \| 1001032 \| 1001625 \| 1 \| \| 22 \| 1001171 \| 1001446 \| 1 \| \| 23 \| 1001585 \| 1001202 \| 1 \| \| 25 \| 1000965 \| 1000963 \| 1 \| \| 26 \| 1000965 \| 1000963 \| 1 \| \| 27 \| 1001294 \| 1002385 \| 1 \| \| 28 \| 1002445 \| 1003152 \| 1 \| \| 29 \| 1002101 \| 1003125 \| 1 \| \| 30 \| 1003194 \| 1000184 \| 1 \| \| 31 \| 1002445 \| 1003152 \| 1 \| \| 32 \| 1001952 \| 1001482 \| 1 \| \| 33 \| 1001051 \| 1001837 \| 1 \| \| 34 \| 1001462 \| 1000242 \| 1 \| \| 35 \| 1001481 \| 1002437 \| 1 \| \| 36 \| 1002445 \| 1003152 \| 1 \| \| 37 \| 1001481 \| 1002437 \| 1 \| \| 38 \| 1003085 \| 1000007 \| 1 \| \| 39 \| 1001622 \| 1001214 \| 1 \| \| 40 \| 1003194 \| 1000467 \| 1 \| \| 41 \| 1003085 \| 1000007 \| 1 \| \| 42 \| 1001358 \| 1001903 \| 1 \| \| 43 \| 1003065 \| 1000273 \| 1 \| \| 44 \| 1002101 \| 1003125 \| 1 \| \| 45 \| 1003065 \| 1000273 \| 1 \| \| 46 \| 1003161 \| 1000182 \| 1 \| \| 47 \| 1001217 \| 1000603 \| 1 \| \| 48 \| 1001217 \| 1000603 \| 1 \| \| 49 \| 1000272 \| 1002475 \| 1 \| \| 50 \| 1000863 \| 1000896 \| 1 \| \| 51 \| 1000788 \| 1001025 \| 1 \| \| 52 \| 1001294 \| 1002385 \| 1 \| \| 53 \| 1002466 \| 1000904 \| 1 \| \| 54 \| 1003047 \| 1000279 \| 1 \| \| 55 \| 1000911 \| 1002163 \| 1 \| \| 56 \| 1002981 \| 1002436 \| 1 \| \| 57 \| 1003065 \| 1000273 \| 1 \| \| 58 \| 1000911 \| 1002163 \| 1 \| \| 59 \| 1000961 \| 1001624 \| 1 \| \| 61 \| 1003194 \| 1000184 \| 1 \| \| 62 \| 1000788 \| 1001025 \| 1 \| \| 63 \| 1000272 \| 1002475 \| 1 \| \| 64 \| 1002781 \| 1001925 \| 1 \| \| 65 \| 1001217 \| 1000603 \| 1 \| \| 66 \| 1001294 \| 1002385 \| 1 \| \| 67 \| 1002781 \| 1001925 \| 1 \| \| 68 \| 1001294 \| 1002385 \| 1 \| \| 69 \| 1001875 \| 1001271 \| 1 \| \| 70 \| 1002262 \| 1001400 \| 1 \| \| 71 \| 1001051 \| 1001837 \| 1 \| \| 72 \| 1001927 \| 1001486 \| 1 \| \| 73 \| 1000574 \| 1001175 \| 1 \| \| 74 \| 1001585 \| 1001202 \| 1 \| \| 75 \| 1003194 \| 1000184 \| 1 \| \| 76 \| 1001875 \| 1002567 \| 1 \| \| 77 \| 1000863 \| 1000896 \| 1 \| \| 78 \| 1002414 \| 1001599 \| 1 \| \| 79 \| 1001051 \| 1001837 \| 1 \| \| 81 \| 1003041 \| 1000292 \| 1 \| \| 82 \| 1003161 \| 1000182 \| 1 \| \| 83 \| 1001051 \| 1001837 \| 1 \| \| 84 \| 1000272 \| 1002475 \| 1 \| \| 85 \| 1000185 \| 1002835 \| 1 \| \| 86 \| 1003173 \| 1000661 \| 1 \| \| 87 \| 1000863 \| 1001983 \| 1 \| \| 88 \| 1002791 \| 1000717 \| 1 \| \| 89 \| 1003173 \| 1000661 \| 1 \| \| 90 \| 1000863 \| 1000896 \| 1 \| \| 91 \| 1000185 \| 1000551 \| 1 \| \| 93 \| 1003161 \| 1000182 \| 1 \| \| 94 \| 1000574 \| 1001175 \| 1 \| \| 95 \| 1000795 \| 1002005 \| 1 \| \| 96 \| 1003194 \| 1000467 \| 1 \| \| 97 \| 1001358 \| 1000806 \| 1 \| \| 98 \| 1003138 \| 1000664 \| 1 \| \| 99 \| 1003173 \| 1000661 \| 1 \| \| 100 \| 1002466 \| 1000904 \| 1 \| \| 101 \| 1000844 \| 1001054 \| 1 \| \| 102 \| 1000911 \| 1002163 \| 1 \| \| 103 \| 1002262 \| 1001400 \| 1 \| \| 104 \| 1001171 \| 1001446 \| 1 \| \| 105 \| 1002781 \| 1001925 \| 1 \| \| 106 \| 1001452 \| 1000152 \| 1 \| \| 107 \| 1003173 \| 1000661 \| 1 \| \| 108 \| 1001462 \| 1000242 \| 1 \| \| 109 \| 1001452 \| 1002526 \| 1 \| \| 110 \| 1003173 \| 1000661 \| 1 \| \| 111 \| 1000541 \| 1002467 \| 1 \| \| 112 \| 1000879 \| 1001052 \| 1 \| \| 113 \| 1000185 \| 1002835 \| 1 \| \| 114 \| 1000541 \| 1002467 \| 1 \| \| 115 \| 1002445 \| 1003152 \| 1 \| \| 116 \| 1000185 \| 1002835 \| 1 \| \| 117 \| 1000126 \| 1002301 \| 1 \| \| 118 \| 1001952 \| 1001482 \| 1 \| \| 119 \| 1002553 \| 1000311 \| 1 \| \| 120 \| 1000961 \| 1001624 \| 1 \| \| 121 \| 1001452 \| 1000152 \| 1 \| \| 122 \| 1001896 \| 1001261 \| 1 \| \| 123 \| 1001452 \| 1000152 \| 1 \| \| 124 \| 1000155 \| 1002303 \| 1 \| \| 125 \| 1001622 \| 1001214 \| 1 \| \| 126 \| 1001452 \| 1002526 \| 1 \| \| 127 \| 1003194 \| 1000467 \| 1 \| \| 128 \| 1001452 \| 1002526 \| 1 \| \| 129 \| 1001193 \| 1000614 \| 1 \| \| 130 \| 1001452 \| 1002526 \| 1 \| \| 131 \| 1000879 \| 1001052 \| 1 \| \| 132 \| 1001622 \| 1001214 \| 1 \| \| 133 \| 1001193 \| 1000614 \| 1 \| \| 134 \| 1001032 \| 1001625 \| 1 \| \| 135 \| 1001481 \| 1002437 \| 1 \| \| 136 \| 1001032 \| 1001625 \| 1 \| \| 137 \| 1000863 \| 1001983 \| 1 \| \| 138 \| 1002781 \| 1001925 \| 1 \| \| 139 \| 1001481 \| 1002437 \| 1 \| \| 140 \| 1003065 \| 1000273 \| 1 \| \| 141 \| 1003085 \| 1000007 \| 1 \| \| 142 \| 1002553 \| 1000311 \| 1 \| \| 143 \| 1001585 \| 1001208 \| 1 \| \| 144 \| 1000155 \| 1002303 \| 1 \| \| 145 \| 1002553 \| 1000311 \| 1 \| \| 146 \| 1002553 \| 1000311 \| 1 \| \| 147 \| 1003194 \| 1000467 \| 1 \| \| 148 \| 1002552 \| 1000919 \| 1 \| \| 149 \| 1003194 \| 1000467 \| 1 \| \| 150 \| 1003194 \| 1000467 \| 1 \| \| 151 \| 1003194 \| 1000467 \| 1 \| \| 152 \| 1000013 \| 1001872 \| 1 \| \| 153 \| 1000013 \| 1001872 \| 1 \| \| 154 \| 1001481 \| 1002437 \| 1 \| \| 155 \| 1001481 \| 1002437 \| 1 \| \| 156 \| 1000541 \| 1002467 \| 1 \| \| 157 \| 1000155 \| 1002303 \| 1 \| \| 158 \| 1000155 \| 1002303 \| 1 \| \| 159 \| 1001622 \| 1001214 \| 1 \| \| 160 \| 1001358 \| 1000806 \| 1 \| \| 161 \| 1003138 \| 1000664 \| 1 \| \| 162 \| 1000126 \| 1002301 \| 1 \| \| 163 \| 1003194 \| 1000467 \| 1 \| \| 164 \| 1001622 \| 1001214 \| 1 \| \| 165 \| 1001622 \| 1001214 \| 1 \| \| 166 \| 1001622 \| 1001214 \| 1 \| \| 168 \| 1000126 \| 1002301 \| 1 \| \| 169 \| 1003041 \| 1000292 \| 1 \| \| 170 \| 1001217 \| 1000603 \| 1 \| \| 171 \| 1003194 \| 1000184 \| 1 \| \| 172 \| 1001585 \| 1001202 \| 1 \| \| 173 \| 1003194 \| 1000184 \| 1 \| \| 174 \| 1003138 \| 1000664 \| 1 \| \| 175 \| 1003138 \| 1000664 \| 1 \| \| 176 \| 1003161 \| 1000182 \| 1 \| \| 177 \| 1001481 \| 1002437 \| 1 \| \| 178 \| 1002101 \| 1003125 \| 1 \| \| 179 \| 1001481 \| 1002437 \| 1 \| \| 180 \| 1002101 \| 1003125 \| 1 \| \| 181 \| 1002466 \| 1000904 \| 1 \| \| 182 \| 1000844 \| 1001054 \| 1 \| \| 183 \| 1000272 \| 1002475 \| 1 \| \| 184 \| 1000844 \| 1001054 \| 1 \| \| 185 \| 1000844 \| 1001054 \| 1 \| \| 186 \| 1000185 \| 1002835 \| 1 \| \| 187 \| 1000272 \| 1002475 \|  \| \| 188 \| 1001481 \| 1002437 \|  \| \| 471 \| 2000661 \| 2001785 \| 2 \| \| 479 \| 2000661 \| 2001785 \| 2 \| \| 487 \| 2000661 \| 2001785 \| 2 \| \| 495 \| 2000661 \| 2001785 \| 2 \| \| 503 \| 2000661 \| 2001785 \| 2 \| \| 511 \| 2000661 \| 2001785 \| 2 \| \| 519 \| 2000661 \| 2001785 \| 2 \| \| 527 \| 2000661 \| 2001785 \| 2 \| \| 535 \| 2000661 \| 2001785 \| 2 \| \| 543 \| 2000661 \| 2001785 \| 2 \| \| 551 \| 2000661 \| 2001785 \| 2 \| \| 559 \| 2000661 \| 2001785 \| 2 \| \| 472 \| 2000661 \| 2001785 \| 2 \| \| 480 \| 2000661 \| 2001785 \| 2 \| \| 488 \| 2000661 \| 2001785 \| 2 \| \| 496 \| 2000375 \| 2000666 \| 2 \| \| 504 \| 2000375 \| 2000666 \| 2 \| \| 512 \| 2000375 \| 2000666 \| 2 \| \| 520 \| 2000375 \| 2000666 \| 2 \| \| 528 \| 2000375 \| 2000666 \| 2 \| \| 536 \| 2000375 \| 2000666 \| 2 \| \| 544 \| 2000375 \| 2000666 \| 2 \| \| 552 \| 2000375 \| 2000666 \| 2 \| \| 560 \| 2000375 \| 2000666 \| 2 \| \| 473 \| 2000375 \| 2000666 \| 2 \| \| 481 \| 2000375 \| 2000666 \| 2 \| \| 497 \| 2000375 \| 2000666 \| 2 \| \| 505 \| 2000375 \| 2000666 \| 2 \| \| 513 \| 2000375 \| 2000666 \| 2 \| \| 521 \| 2002023 \| 2000902 \| 2 \| \| 529 \| 2002023 \| 2000902 \| 2 \| \| 537 \| 2002023 \| 2000902 \| 2 \| \| 545 \| 2002023 \| 2000902 \| 2 \| \| 553 \| 2002023 \| 2000902 \| 2 \| \| 561 \| 2002023 \| 2000902 \| 2 \| \| 474 \| 2002023 \| 2000902 \| 2 \| \| 482 \| 2002023 \| 2000902 \| 2 \| \| 490 \| 2002023 \| 2000902 \| 2 \| \| 498 \| 2002023 \| 2000902 \| 2 \| \| 506 \| 2002023 \| 2000902 \| 2 \| \| 514 \| 2002023 \| 2000902 \| 2 \| \| 522 \| 2002023 \| 2000902 \| 2 \| \| 530 \| 2002023 \| 2000902 \| 2 \| \| 538 \| 2002023 \| 2000902 \| 2 \| \| 546 \| 2002601 \| 2000267 \| 2 \| \| 554 \| 2002601 \| 2000267 \| 2 \| \| 562 \| 2002601 \| 2000267 \| 2 \| \| 475 \| 2002601 \| 2000267 \| 2 \| \| 483 \| 2002601 \| 2000267 \| 2 \| \| 491 \| 2002601 \| 2000267 \| 2 \| \| 499 \| 2002601 \| 2000267 \| 2 \| \| 507 \| 2002601 \| 2000267 \| 2 \| \| 515 \| 2002601 \| 2000267 \| 2 \| \| 523 \| 2002601 \| 2000267 \| 2 \| \| 531 \| 2002601 \| 2000267 \| 2 \| \| 539 \| 2002601 \| 2000267 \| 2 \| \| 547 \| 2002601 \| 2000267 \| 2 \| \| 555 \| 2002601 \| 2000267 \| 2 \| \| 563 \| 2002601 \| 2000267 \| 2 \| \| 493 \| 2000439 \| 2001807 \| 2 \| \| 501 \| 2000439 \| 2001807 \| 2 \| \| 509 \| 2000439 \| 2001807 \| 2 \| \| 517 \| 2000439 \| 2001807 \| 2 \| \| 525 \| 2000439 \| 2001807 \| 2 \| \| 533 \| 2000439 \| 2001807 \| 2 \| \| 541 \| 2000439 \| 2001807 \| 2 \| \| 549 \| 2000439 \| 2001807 \| 2 \| \| 557 \| 2000439 \| 2001807 \| 2 \| \| 478 \| 2000439 \| 2001807 \| 2 \| \| 486 \| 2000439 \| 2001807 \| 2 \| \| 494 \| 2000439 \| 2001807 \| 2 \| \| 502 \| 2000439 \| 2001807 \| 2 \| \| 605 \| 2002112 \| 2002367 \| 2 \| \| 597 \| 2002112 \| 2002367 \| 2 \| \| 589 \| 2002112 \| 2002367 \| 2 \| \| 581 \| 2002112 \| 2002367 \| 2 \| \| 573 \| 2002112 \| 2002367 \| 2 \| \| 565 \| 2002112 \| 2002367 \| 2 \| \| 448 \| 2002112 \| 2002367 \| 2 \| \| 189 \| 2002112 \| 2002367 \| 2 \| \| 197 \| 2002112 \| 2002367 \| 2 \| \| 205 \| 2002112 \| 2002367 \| 2 \| \| 213 \| 2002112 \| 2002367 \| 2 \| \| 221 \| 2002112 \| 2002367 \| 2 \| \| 229 \| 2002112 \| 2002367 \| 2 \| \| 283 \| 2002112 \| 2002367 \| 2 \| \| 291 \| 2002112 \| 2002367 \| 2 \| \| 299 \| 2000580 \| 2000671 \| 2 \| \| 307 \| 2000580 \| 2000671 \| 2 \| \| 315 \| 2000580 \| 2000671 \| 2 \| \| 323 \| 2000580 \| 2000671 \| 2 \| \| 331 \| 2000580 \| 2000671 \| 2 \| \| 339 \| 2000580 \| 2000671 \| 2 \| \| 347 \| 2000580 \| 2000671 \| 2 \| \| 355 \| 2000580 \| 2000671 \| 2 \| \| 363 \| 2000580 \| 2000671 \| 2 \| \| 371 \| 2000580 \| 2000671 \| 2 \| \| 284 \| 2000580 \| 2000671 \| 2 \| \| 292 \| 2000580 \| 2000671 \| 2 \| \| 300 \| 2000580 \| 2000671 \| 2 \| \| 308 \| 2000580 \| 2000671 \| 2 \| \| 316 \| 2000580 \| 2000671 \| 2 \| \| 324 \| 2000117 \| 2002282 \| 2 \| \| 332 \| 2000117 \| 2002282 \| 2 \| \| 340 \| 2000117 \| 2002282 \| 2 \| \| 348 \| 2000117 \| 2002282 \| 2 \| \| 356 \| 2000117 \| 2002282 \| 2 \| \| 364 \| 2000117 \| 2002282 \| 2 \| \| 372 \| 2000117 \| 2002282 \| 2 \| \| 293 \| 2000117 \| 2002282 \| 2 \| \| 301 \| 2000117 \| 2002282 \| 2 \| \| 309 \| 2000117 \| 2002282 \| 2 \| \| 317 \| 2000117 \| 2002282 \| 2 \| \| 325 \| 2000117 \| 2002282 \| 2 \| \| 333 \| 2000117 \| 2002282 \| 2 \| \| 341 \| 2000117 \| 2002282 \| 2 \| \| 349 \| 2000117 \| 2002282 \| 2 \| \| 357 \| 2000284 \| 2002680 \| 2 \| \| 285 \| 2000284 \| 2002680 \| 2 \| \| 365 \| 2000284 \| 2002680 \| 2 \| \| 373 \| 2000284 \| 2002680 \| 2 \| \| 286 \| 2000284 \| 2002680 \| 2 \| \| 294 \| 2000284 \| 2002680 \| 2 \| \| 310 \| 2000284 \| 2002680 \| 2 \| \| 318 \| 2000284 \| 2002680 \| 2 \| \| 326 \| 2000284 \| 2002680 \| 2 \| \| 334 \| 2000284 \| 2002680 \| 2 \| \| 342 \| 2000284 \| 2002680 \| 2 \| \| 350 \| 2000284 \| 2002680 \| 2 \| \| 358 \| 2000284 \| 2002680 \| 2 \| \| 366 \| 2000284 \| 2002680 \| 2 \| \| 374 \| 2002617 \| 2000976 \| 2 \| \| 287 \| 2002617 \| 2000976 \| 2 \| \| 295 \| 2002617 \| 2000976 \| 2 \| \| 302 \| 2002617 \| 2000976 \| 2 \| \| 311 \| 2002617 \| 2000976 \| 2 \| \| 319 \| 2002617 \| 2000976 \| 2 \| \| 327 \| 2002617 \| 2000976 \| 2 \| \| 335 \| 2002617 \| 2000976 \| 2 \| \| 343 \| 2002617 \| 2000976 \| 2 \| \| 351 \| 2002617 \| 2000976 \| 2 \| \| 359 \| 2002617 \| 2000976 \| 2 \| \| 367 \| 2002617 \| 2000976 \| 2 \| \| 375 \| 2002617 \| 2000976 \| 2 \| \| 288 \| 2002617 \| 2000976 \| 2 \| \| 296 \| 2002617 \| 2000976 \| 2 \| \| 303 \| 2002681 \| 2002152 \| 2 \| \| 312 \| 2002681 \| 2002152 \| 2 \| \| 320 \| 2002681 \| 2002152 \| 2 \| \| 328 \| 2002681 \| 2002152 \| 2 \| \| 336 \| 2002681 \| 2002152 \| 2 \| \| 344 \| 2002681 \| 2002152 \| 2 \| \| 352 \| 2002681 \| 2002152 \| 2 \| \| 360 \| 2002681 \| 2002152 \| 2 \| \| 368 \| 2002681 \| 2002152 \| 2 \| \| 376 \| 2002681 \| 2002152 \| 2 \| \| 289 \| 2002681 \| 2002152 \| 2 \| \| 297 \| 2002681 \| 2002152 \| 2 \| \| 304 \| 2002681 \| 2002152 \| 2 \| \| 313 \| 2002681 \| 2002152 \| 2 \| \| 321 \| 2002681 \| 2002152 \| 2 \| \| 329 \| 2001256 \| 2002725 \| 2 \| \| 337 \| 2001256 \| 2002725 \| 2 \| \| 345 \| 2001256 \| 2002725 \| 2 \| \| 353 \| 2001256 \| 2002725 \| 2 \| \| 361 \| 2001256 \| 2002725 \| 2 \| \| 369 \| 2001256 \| 2002725 \| 2 \| \| 456 \| 2001256 \| 2002725 \| 2 \| \| 290 \| 2001256 \| 2002725 \| 2 \| \| 298 \| 2001256 \| 2002725 \| 2 \| \| 305 \| 2001256 \| 2002725 \| 2 \| \| 314 \| 2001256 \| 2002725 \| 2 \| \| 322 \| 2001256 \| 2002725 \| 2 \| \| 330 \| 2001256 \| 2002725 \| 2 \| \| 338 \| 2001256 \| 2002725 \| 2 \| \| 346 \| 2001256 \| 2002725 \| 2 \| \| 630 \| 2002151 \| 2001013 \| 2 \| \| 622 \| 2002151 \| 2001013 \| 2 \| \| 614 \| 2002151 \| 2001013 \| 2 \| \| 606 \| 2002151 \| 2001013 \| 2 \| \| 598 \| 2002151 \| 2001013 \| 2 \| \| 590 \| 2002151 \| 2001013 \| 2 \| \| 582 \| 2002151 \| 2001013 \| 2 \| \| 574 \| 2002151 \| 2001013 \| 2 \| \| 566 \| 2002151 \| 2001013 \| 2 \| \| 653 \| 2002151 \| 2001013 \| 2 \| \| 645 \| 2002151 \| 2001013 \| 2 \| \| 637 \| 2002151 \| 2001013 \| 2 \| \| 629 \| 2002151 \| 2001013 \| 2 \| \| 621 \| 2002151 \| 2001013 \| 2 \| \| 613 \| 2002151 \| 2001013 \| 2 \| \| 655 \| 2000776 \| 2000839 \| 2 \| \| 647 \| 2000776 \| 2000839 \| 2 \| \| 639 \| 2000776 \| 2000839 \| 2 \| \| 631 \| 2000776 \| 2000839 \| 2 \| \| 623 \| 2000776 \| 2000839 \| 2 \| \| 615 \| 2000776 \| 2000839 \| 2 \| \| 607 \| 2000776 \| 2000839 \| 2 \| \| 599 \| 2000776 \| 2000839 \| 2 \| \| 591 \| 2000776 \| 2000839 \| 2 \| \| 575 \| 2000776 \| 2000839 \| 2 \| \| 567 \| 2000776 \| 2000839 \| 2 \| \| 654 \| 2000776 \| 2000839 \| 2 \| \| 646 \| 2000776 \| 2000839 \| 2 \| \| 638 \| 2000776 \| 2000839 \| 2 \| \| 585 \| 2000776 \| 2000041 \| 2 \| \| 577 \| 2000776 \| 2000041 \| 2 \| \| 569 \| 2000776 \| 2000041 \| 2 \| \| 656 \| 2000776 \| 2000041 \| 2 \| \| 648 \| 2000776 \| 2000041 \| 2 \| \| 640 \| 2000776 \| 2000041 \| 2 \| \| 632 \| 2000776 \| 2000041 \| 2 \| \| 624 \| 2000776 \| 2000041 \| 2 \| \| 616 \| 2000776 \| 2000041 \| 2 \| \| 608 \| 2000776 \| 2000041 \| 2 \| \| 600 \| 2000776 \| 2000041 \| 2 \| \| 592 \| 2000776 \| 2000041 \| 2 \| \| 584 \| 2000776 \| 2000041 \| 2 \| \| 576 \| 2000776 \| 2000041 \| 2 \| \| 568 \| 2000776 \| 2000041 \| 2 \| \| 610 \| 2001021 \| 2002111 \| 2 \| \| 602 \| 2001021 \| 2002111 \| 2 \| \| 594 \| 2001021 \| 2002111 \| 2 \| \| 586 \| 2001021 \| 2002111 \| 2 \| \| 578 \| 2001021 \| 2002111 \| 2 \| \| 570 \| 2001021 \| 2002111 \| 2 \| \| 657 \| 2001021 \| 2002111 \| 2 \| \| 649 \| 2001021 \| 2002111 \| 2 \| \| 641 \| 2001021 \| 2002111 \| 2 \| \| 633 \| 2001021 \| 2002111 \| 2 \| \| 625 \| 2001021 \| 2002111 \| 2 \| \| 617 \| 2001021 \| 2002111 \| 2 \| \| 609 \| 2001021 \| 2002111 \| 2 \| \| 601 \| 2001021 \| 2002111 \| 2 \| \| 593 \| 2001021 \| 2002111 \| 2 \| \| 635 \| 2000896 \| 2001027 \| 2 \| \| 627 \| 2000896 \| 2001027 \| 2 \| \| 619 \| 2000896 \| 2001027 \| 2 \| \| 611 \| 2000896 \| 2001027 \| 2 \| \| 603 \| 2000896 \| 2001027 \| 2 \| \| 595 \| 2000896 \| 2001027 \| 2 \| \| 587 \| 2000896 \| 2001027 \| 2 \| \| 579 \| 2000896 \| 2001027 \| 2 \| \| 571 \| 2000896 \| 2001027 \| 2 \| \| 658 \| 2000896 \| 2001027 \| 2 \| \| 650 \| 2000896 \| 2001027 \| 2 \| \| 642 \| 2000896 \| 2001027 \| 2 \| \| 634 \| 2000896 \| 2001027 \| 2 \| \| 626 \| 2000896 \| 2001027 \| 2 \| \| 618 \| 2000896 \| 2001027 \| 2 \| \| 652 \| 2000170 \| 2000231 \| 2 \| \| 644 \| 2000170 \| 2000231 \| 2 \| \| 636 \| 2000170 \| 2000231 \| 2 \| \| 628 \| 2000170 \| 2000231 \| 2 \| \| 620 \| 2000170 \| 2000231 \| 2 \| \| 612 \| 2000170 \| 2000231 \| 2 \| \| 604 \| 2000170 \| 2000231 \| 2 \| \| 596 \| 2000170 \| 2000231 \| 2 \| \| 588 \| 2000170 \| 2000231 \| 2 \| \| 580 \| 2000170 \| 2000231 \| 2 \| \| 572 \| 2000170 \| 2000231 \| 2 \| \| 432 \| 2000170 \| 2000231 \| 2 \| \| 651 \| 2000170 \| 2000231 \| 2 \| \| 643 \| 2000170 \| 2000231 \| 2 \| \| 254 \| 2000664 \| 2002361 \| 2 \| \| 246 \| 2000664 \| 2002361 \| 2 \| \| 238 \| 2000664 \| 2002361 \| 2 \| \| 230 \| 2000664 \| 2002361 \| 2 \| \| 222 \| 2000664 \| 2002361 \| 2 \| \| 214 \| 2000664 \| 2002361 \| 2 \| \| 206 \| 2000664 \| 2002361 \| 2 \| \| 198 \| 2000664 \| 2002361 \| 2 \| \| 190 \| 2000664 \| 2002361 \| 2 \| \| 277 \| 2000664 \| 2002361 \| 2 \| \| 269 \| 2000664 \| 2002361 \| 2 \| \| 261 \| 2000664 \| 2002361 \| 2 \| \| 253 \| 2000664 \| 2002361 \| 2 \| \| 245 \| 2000664 \| 2002361 \| 2 \| \| 237 \| 2000664 \| 2002361 \| 2 \| \| 279 \| 2000266 \| 2000750 \| 2 \| \| 271 \| 2000266 \| 2000750 \| 2 \| \| 263 \| 2000266 \| 2000750 \| 2 \| \| 255 \| 2000266 \| 2000750 \| 2 \| \| 247 \| 2000266 \| 2000750 \| 2 \| \| 239 \| 2000266 \| 2000750 \| 2 \| \| 231 \| 2000266 \| 2000750 \| 2 \| \| 223 \| 2000266 \| 2000750 \| 2 \| \| 215 \| 2000266 \| 2000750 \| 2 \| \| 207 \| 2000266 \| 2000750 \| 2 \| \| 199 \| 2000266 \| 2000750 \| 2 \| \| 191 \| 2000266 \| 2000750 \| 2 \| \| 278 \| 2000266 \| 2000750 \| 2 \| \| 270 \| 2000266 \| 2000750 \| 2 \| \| 262 \| 2000266 \| 2000750 \| 2 \| \| 209 \| 2000296 \| 2001761 \| 2 \| \| 201 \| 2000296 \| 2001761 \| 2 \| \| 193 \| 2000296 \| 2001761 \| 2 \| \| 280 \| 2000296 \| 2001761 \| 2 \| \| 272 \| 2000296 \| 2001761 \| 2 \| \| 264 \| 2000296 \| 2001761 \| 2 \| \| 256 \| 2000296 \| 2001761 \| 2 \| \| 248 \| 2000296 \| 2001761 \| 2 \| \| 240 \| 2000296 \| 2001761 \| 2 \| \| 232 \| 2000296 \| 2001761 \| 2 \| \| 224 \| 2000296 \| 2001761 \| 2 \| \| 216 \| 2000296 \| 2001761 \| 2 \| \| 208 \| 2000296 \| 2001761 \| 2 \| \| 200 \| 2000296 \| 2001761 \| 2 \| \| 192 \| 2000296 \| 2001761 \| 2 \| \| 234 \| 2001874 \| 2002620 \| 2 \| \| 226 \| 2001874 \| 2002620 \| 2 \| \| 218 \| 2001874 \| 2002620 \| 2 \| \| 210 \| 2001874 \| 2002620 \| 2 \| \| 202 \| 2001874 \| 2002620 \| 2 \| \| 194 \| 2001874 \| 2002620 \| 2 \| \| 281 \| 2001874 \| 2002620 \| 2 \| \| 273 \| 2001874 \| 2002620 \| 2 \| \| 265 \| 2001874 \| 2002620 \| 2 \| \| 257 \| 2001874 \| 2002620 \| 2 \| \| 249 \| 2001874 \| 2002620 \| 2 \| \| 241 \| 2001874 \| 2002620 \| 2 \| \| 233 \| 2001874 \| 2002620 \| 2 \| \| 225 \| 2001874 \| 2002620 \| 2 \| \| 217 \| 2001874 \| 2002620 \| 2 \| \| 259 \| 2000874 \| 2000258 \| 2 \| \| 251 \| 2000874 \| 2000258 \| 2 \| \| 243 \| 2000874 \| 2000258 \| 2 \| \| 235 \| 2000874 \| 2000258 \| 2 \| \| 227 \| 2000874 \| 2000258 \| 2 \| \| 219 \| 2000874 \| 2000258 \| 2 \| \| 211 \| 2000874 \| 2000258 \| 2 \| \| 203 \| 2000874 \| 2000258 \| 2 \| \| 195 \| 2000874 \| 2000258 \| 2 \| \| 282 \| 2000874 \| 2000258 \| 2 \| \| 274 \| 2000874 \| 2000258 \| 2 \| \| 266 \| 2000874 \| 2000258 \| 2 \| \| 258 \| 2000874 \| 2000258 \| 2 \| \| 250 \| 2000874 \| 2000258 \| 2 \| \| 242 \| 2000874 \| 2000258 \| 2 \| \| 276 \| 2000266 \| 2000750 \| 2 \| \| 268 \| 2000266 \| 2000750 \| 2 \| \| 260 \| 2000266 \| 2000750 \| 2 \| \| 252 \| 2000266 \| 2000750 \| 2 \| \| 244 \| 2000266 \| 2000750 \| 2 \| \| 236 \| 2000266 \| 2000750 \| 2 \| \| 228 \| 2000266 \| 2000750 \| 2 \| \| 220 \| 2000266 \| 2000750 \| 2 \| \| 212 \| 2000266 \| 2000750 \| 2 \| \| 204 \| 2000266 \| 2000750 \| 2 \| \| 196 \| 2000266 \| 2000750 \| 2 \| \| 440 \| 2000266 \| 2000750 \| 2 \| \| 275 \| 2000266 \| 2000750 \| 2 \| \| 267 \| 2000266 \| 2000750 \| 2 \| \| 377 \| 2000378 \| 2000539 \| 2 \| \| 385 \| 2000378 \| 2000539 \| 2 \| \| 393 \| 2000378 \| 2000539 \| 2 \| \| 401 \| 2000378 \| 2000539 \| 2 \| \| 409 \| 2000378 \| 2000539 \| 2 \| \| 417 \| 2000378 \| 2000539 \| 2 \| \| 425 \| 2000378 \| 2000539 \| 2 \| \| 433 \| 2000378 \| 2000539 \| 2 \| \| 441 \| 2000378 \| 2000539 \| 2 \| \| 449 \| 2000378 \| 2000539 \| 2 \| \| 457 \| 2000378 \| 2000539 \| 2 \| \| 465 \| 2000378 \| 2000539 \| 2 \| \| 378 \| 2000378 \| 2000539 \| 2 \| \| 386 \| 2000378 \| 2000539 \| 2 \| \| 394 \| 2000378 \| 2000539 \| 2 \| \| 402 \| 2001590 \| 2001207 \| 2 \| \| 410 \| 2001590 \| 2001207 \| 2 \| \| 418 \| 2001590 \| 2001207 \| 2 \| \| 426 \| 2001590 \| 2001207 \| 2 \| \| 442 \| 2001590 \| 2001207 \| 2 \| \| 450 \| 2001590 \| 2001207 \| 2 \| \| 458 \| 2001590 \| 2001207 \| 2 \| \| 466 \| 2001590 \| 2001207 \| 2 \| \| 379 \| 2001590 \| 2001207 \| 2 \| \| 387 \| 2001590 \| 2001207 \| 2 \| \| 395 \| 2001590 \| 2001207 \| 2 \| \| 403 \| 2001590 \| 2001207 \| 2 \| \| 411 \| 2001590 \| 2001207 \| 2 \| \| 419 \| 2001590 \| 2001207 \| 2 \| \| 427 \| 2000747 \| 2001205 \| 2 \| \| 435 \| 2000747 \| 2001205 \| 2 \| \| 443 \| 2000747 \| 2001205 \| 2 \| \| 451 \| 2000747 \| 2001205 \| 2 \| \| 459 \| 2000747 \| 2001205 \| 2 \| \| 467 \| 2000747 \| 2001205 \| 2 \| \| 380 \| 2000747 \| 2001205 \| 2 \| \| 396 \| 2000747 \| 2001205 \| 2 \| \| 354 \| 2000747 \| 2001205 \| 2 \| \| 362 \| 2000747 \| 2001205 \| 2 \| \| 370 \| 2000747 \| 2001205 \| 2 \| \| 306 \| 2000747 \| 2001205 \| 2 \| \| 510 \| 2002106 \| 2000637 \| 2 \| \| 518 \| 2002106 \| 2000637 \| 2 \| \| 526 \| 2002106 \| 2000637 \| 2 \| \| 534 \| 2002106 \| 2000637 \| 2 \| \| 542 \| 2002106 \| 2000637 \| 2 \| \| 550 \| 2002106 \| 2000637 \| 2 \| \| 558 \| 2002106 \| 2000637 \| 2 \| \| 464 \| 2002106 \| 2000637 \| 2 \| \| 404 \| 2002106 \| 2000637 \| 2 \| \| 412 \| 2002106 \| 2000637 \| 2 \| \| 420 \| 2002106 \| 2000637 \| 2 \| \| 428 \| 2002106 \| 2000637 \| 2 \| \| 436 \| 2002106 \| 2000637 \| 2 \| \| 444 \| 2002106 \| 2000637 \| 2 \| \| 452 \| 2002106 \| 2000637 \| 2 \| \| 468 \| 2000063 \| 2001620 \| 2 \| \| 381 \| 2000063 \| 2001620 \| 2 \| \| 389 \| 2000063 \| 2001620 \| 2 \| \| 397 \| 2000063 \| 2001620 \| 2 \| \| 405 \| 2000063 \| 2001620 \| 2 \| \| 413 \| 2000063 \| 2001620 \| 2 \| \| 421 \| 2000063 \| 2001620 \| 2 \| \| 429 \| 2000063 \| 2001620 \| 2 \| \| 437 \| 2000063 \| 2001620 \| 2 \| \| 445 \| 2000063 \| 2001620 \| 2 \| \| 453 \| 2000063 \| 2001620 \| 2 \| \| 461 \| 2000063 \| 2001620 \| 2 \| \| 469 \| 2000063 \| 2001620 \| 2 \| \| 382 \| 2000063 \| 2001620 \| 2 \| \| 390 \| 2000747 \| 2001870 \| 2 \| \| 398 \| 2000747 \| 2001870 \| 2 \| \| 406 \| 2000747 \| 2001870 \| 2 \| \| 414 \| 2000747 \| 2001870 \| 2 \| \| 422 \| 2000747 \| 2001870 \| 2 \| \| 430 \| 2000747 \| 2001870 \| 2 \| \| 438 \| 2000747 \| 2001870 \| 2 \| \| 446 \| 2000747 \| 2001870 \| 2 \| \| 454 \| 2000747 \| 2001870 \| 2 \| \| 462 \| 2000747 \| 2001870 \| 2 \| \| 470 \| 2000747 \| 2001870 \| 2 \| \| 383 \| 2000747 \| 2001870 \| 2 \| \| 391 \| 2000747 \| 2001870 \| 2 \| \| 399 \| 2000747 \| 2001870 \| 2 \| \| 407 \| 2000747 \| 2001870 \| 2 \| \| 424 \| 2002161 \| 2000775 \| 2 \| \| 416 \| 2002161 \| 2000775 \| 2 \| \| 408 \| 2002161 \| 2000775 \| 2 \| \| 400 \| 2002161 \| 2000775 \| 2 \| \| 392 \| 2002161 \| 2000775 \| 2 \| \| 384 \| 2002161 \| 2000775 \| 2 \| \| 463 \| 2002161 \| 2000775 \| 2 \| \| 455 \| 2002161 \| 2000775 \| 2 \| \| 447 \| 2002161 \| 2000775 \| 2 \| \| 439 \| 2002161 \| 2000775 \| 2 \| \| 431 \| 2002161 \| 2000775 \| 2 \| \| 423 \| 2002161 \| 2000775 \| 2 \| \| 415 \| 2002161 \| 2000775 \| 2 \| \| 476 \| 2000285 \| 2001758 \| 2 \| \| 484 \| 2000285 \| 2001758 \| 2 \| \| 492 \| 2000285 \| 2001758 \| 2 \| \| 500 \| 2000285 \| 2001758 \| 2 \| \| 508 \| 2000285 \| 2001758 \| 2 \| \| 516 \| 2000285 \| 2001758 \| 2 \| \| 524 \| 2000285 \| 2001758 \| 2 \| \| 532 \| 2000285 \| 2001758 \| 2 \| \| 540 \| 2000285 \| 2001758 \| 2 \| \| 548 \| 2000285 \| 2001758 \| 2 \| \| 556 \| 2000285 \| 2001758 \| 2 \| \| 564 \| 2000285 \| 2001758 \| 2 \| \| 477 \| 2000285 \| 2001758 \| 2 \| \| 485 \| 2000285 \| 2001758 \| 2 \| |  |  |  |
| --- | --- | --- | --- | --- | --- | --- | --- | --- | --- | --- | --- | --- | --- | --- | --- | --- | --- | --- | --- | --- | --- | --- | --- | --- | --- | --- | --- | --- | --- | --- | --- | --- | --- | --- | --- | --- | --- | --- | --- | --- | --- | --- | --- | --- | --- | --- | --- | --- | --- | --- | --- | --- | --- | --- | --- | --- | --- | --- | --- | --- | --- | --- | --- | --- | --- | --- | --- | --- | --- | --- | --- | --- | --- | --- | --- | --- | --- | --- | --- | --- | --- | --- | --- | --- | --- | --- | --- | --- | --- | --- | --- | --- | --- | --- | --- | --- | --- | --- | --- | --- | --- | --- | --- | --- | --- | --- | --- | --- | --- | --- | --- | --- | --- | --- | --- | --- | --- | --- | --- | --- | --- | --- | --- | --- | --- | --- | --- | --- | --- | --- | --- | --- | --- | --- | --- | --- | --- | --- | --- | --- | --- | --- | --- | --- | --- | --- | --- | --- | --- | --- | --- | --- | --- | --- | --- | --- | --- | --- | --- | --- | --- | --- | --- | --- | --- | --- | --- | --- | --- | --- | --- | --- | --- | --- | --- | --- | --- | --- | --- | --- | --- | --- | --- | --- | --- | --- | --- | --- | --- | --- | --- | --- | --- | --- | --- | --- | --- | --- | --- | --- | --- | --- | --- | --- | --- | --- | --- | --- | --- | --- | --- | --- | --- | --- | --- | --- | --- | --- | --- | --- | --- | --- | --- | --- | --- | --- | --- | --- | --- | --- | --- | --- | --- | --- | --- | --- | --- | --- | --- | --- | --- | --- | --- | --- | --- | --- | --- | --- | --- | --- | --- | --- | --- | --- | --- | --- | --- | --- | --- | --- | --- | --- | --- | --- | --- | --- | --- | --- | --- | --- | --- | --- | --- | --- | --- | --- | --- | --- | --- | --- | --- | --- | --- | --- | --- | --- | --- | --- | --- | --- | --- | --- | --- | --- | --- | --- | --- | --- | --- | --- | --- | --- | --- | --- | --- | --- | --- | --- | --- | --- | --- | --- | --- | --- | --- | --- | --- | --- | --- | --- | --- | --- | --- | --- | --- | --- | --- | --- | --- | --- | --- | --- | --- | --- | --- | --- | --- | --- | --- | --- | --- | --- | --- | --- | --- | --- | --- | --- | --- | --- | --- | --- | --- | --- | --- | --- | --- | --- | --- | --- | --- | --- | --- | --- | --- | --- | --- | --- | --- | --- | --- | --- | --- | --- | --- | --- | --- | --- | --- | --- | --- | --- | --- | --- | --- | --- | --- | --- | --- | --- | --- | --- | --- | --- | --- | --- | --- | --- | --- | --- | --- | --- | --- | --- | --- | --- | --- | --- | --- | --- | --- | --- | --- | --- | --- | --- | --- | --- | --- | --- | --- | --- | --- | --- | --- | --- | --- | --- | --- | --- | --- | --- | --- | --- | --- | --- | --- | --- | --- | --- | --- | --- | --- | --- | --- | --- | --- | --- | --- | --- | --- | --- | --- | --- | --- | --- | --- | --- | --- | --- | --- | --- | --- | --- | --- | --- | --- | --- | --- | --- | --- | --- | --- | --- | --- | --- | --- | --- | --- | --- | --- | --- | --- | --- | --- | --- | --- | --- | --- | --- | --- | --- | --- | --- | --- | --- | --- | --- | --- | --- | --- | --- | --- | --- | --- | --- | --- | --- | --- | --- | --- | --- | --- | --- | --- | --- | --- | --- | --- | --- | --- | --- | --- | --- | --- | --- | --- | --- | --- | --- | --- | --- | --- | --- | --- | --- | --- | --- | --- | --- | --- | --- | --- | --- | --- | --- | --- | --- | --- | --- | --- | --- | --- | --- | --- | --- | --- | --- | --- | --- | --- | --- | --- | --- | --- | --- | --- | --- | --- | --- | --- | --- | --- | --- | --- | --- | --- | --- | --- | --- | --- | --- | --- | --- | --- | --- | --- | --- | --- | --- | --- | --- | --- | --- | --- | --- | --- | --- | --- | --- | --- | --- | --- | --- | --- | --- | --- | --- | --- | --- | --- | --- | --- | --- | --- | --- | --- | --- | --- | --- | --- | --- | --- | --- | --- | --- | --- | --- | --- | --- | --- | --- | --- | --- | --- | --- | --- | --- | --- | --- | --- | --- | --- | --- | --- | --- | --- | --- | --- | --- | --- | --- | --- | --- | --- | --- | --- | --- | --- | --- | --- | --- | --- | --- | --- | --- | --- | --- | --- | --- | --- | --- | --- | --- | --- | --- | --- | --- | --- | --- | --- | --- | --- | --- | --- | --- | --- | --- | --- | --- | --- | --- | --- | --- | --- | --- | --- | --- | --- | --- | --- | --- | --- | --- | --- | --- | --- | --- | --- | --- | --- | --- | --- | --- | --- | --- | --- | --- | --- | --- | --- | --- | --- | --- | --- | --- | --- | --- | --- | --- | --- | --- | --- | --- | --- | --- | --- | --- | --- | --- | --- | --- | --- | --- | --- | --- | --- | --- | --- | --- | --- | --- | --- | --- | --- | --- | --- | --- | --- | --- | --- | --- | --- | --- | --- | --- | --- | --- | --- | --- | --- | --- | --- | --- | --- | --- | --- | --- | --- | --- | --- | --- | --- | --- | --- | --- | --- | --- | --- | --- | --- | --- | --- | --- | --- | --- | --- | --- | --- | --- | --- | --- | --- | --- | --- | --- | --- | --- | --- | --- | --- | --- | --- | --- | --- | --- | --- | --- | --- | --- | --- | --- | --- | --- | --- | --- | --- | --- | --- | --- | --- | --- | --- | --- | --- | --- | --- | --- | --- | --- | --- | --- | --- | --- | --- | --- | --- | --- | --- | --- | --- | --- | --- | --- | --- | --- | --- | --- | --- | --- | --- | --- | --- | --- | --- | --- | --- | --- | --- | --- | --- | --- | --- | --- | --- | --- | --- | --- | --- | --- | --- | --- | --- | --- | --- | --- | --- | --- | --- | --- | --- | --- | --- | --- | --- | --- | --- | --- | --- | --- | --- | --- | --- | --- | --- | --- | --- | --- | --- | --- | --- | --- | --- | --- | --- | --- | --- | --- | --- | --- | --- | --- | --- | --- | --- | --- | --- | --- | --- | --- | --- | --- | --- | --- | --- | --- | --- | --- | --- | --- | --- | --- | --- | --- | --- | --- | --- | --- | --- | --- | --- | --- | --- | --- | --- | --- | --- | --- | --- | --- | --- | --- | --- | --- | --- | --- | --- | --- | --- | --- | --- | --- | --- | --- | --- | --- | --- | --- | --- | --- | --- | --- | --- | --- | --- | --- | --- | --- | --- | --- | --- | --- | --- | --- | --- | --- | --- | --- | --- | --- | --- | --- | --- | --- | --- | --- | --- | --- | --- | --- | --- | --- | --- | --- | --- | --- | --- | --- | --- | --- | --- | --- | --- | --- | --- | --- | --- | --- | --- | --- | --- | --- | --- | --- | --- | --- | --- | --- | --- | --- | --- | --- | --- | --- | --- | --- | --- | --- | --- | --- | --- | --- | --- | --- | --- | --- | --- | --- | --- | --- | --- | --- | --- | --- | --- | --- | --- | --- | --- | --- | --- | --- | --- | --- | --- | --- | --- | --- | --- | --- | --- | --- | --- | --- | --- | --- | --- | --- | --- | --- | --- | --- | --- | --- | --- | --- | --- | --- | --- | --- | --- | --- | --- | --- | --- | --- | --- | --- | --- | --- | --- | --- | --- | --- | --- | --- | --- | --- | --- | --- | --- | --- | --- | --- | --- | --- | --- | --- | --- | --- | --- | --- | --- | --- | --- | --- | --- | --- | --- | --- | --- | --- | --- | --- | --- | --- | --- | --- | --- | --- | --- | --- | --- | --- | --- | --- | --- | --- | --- | --- | --- | --- | --- | --- | --- | --- | --- | --- | --- | --- | --- | --- | --- | --- | --- | --- | --- | --- | --- | --- | --- | --- | --- | --- | --- | --- | --- | --- | --- | --- | --- | --- | --- | --- | --- | --- | --- | --- | --- | --- | --- | --- | --- | --- | --- | --- | --- | --- | --- | --- | --- | --- | --- | --- | --- | --- | --- | --- | --- | --- | --- | --- | --- | --- | --- | --- | --- | --- | --- | --- | --- | --- | --- | --- | --- | --- | --- | --- | --- | --- | --- | --- | --- | --- | --- | --- | --- | --- | --- | --- | --- | --- | --- | --- | --- | --- | --- | --- | --- | --- | --- | --- | --- | --- | --- | --- | --- | --- | --- | --- | --- | --- | --- | --- | --- | --- | --- | --- | --- | --- | --- | --- | --- | --- | --- | --- | --- | --- | --- | --- | --- | --- | --- | --- | --- | --- | --- | --- | --- | --- | --- | --- | --- | --- | --- | --- | --- | --- | --- | --- | --- | --- | --- | --- | --- | --- | --- | --- | --- | --- | --- | --- | --- | --- | --- | --- | --- | --- | --- | --- | --- | --- | --- | --- | --- | --- | --- | --- | --- | --- | --- | --- | --- | --- | --- | --- | --- | --- | --- | --- | --- | --- | --- | --- | --- | --- | --- | --- | --- | --- | --- | --- | --- | --- | --- | --- | --- | --- | --- | --- | --- | --- | --- | --- | --- | --- | --- | --- | --- | --- | --- | --- | --- | --- | --- | --- | --- | --- | --- | --- | --- | --- | --- | --- | --- | --- | --- | --- | --- | --- | --- | --- | --- | --- | --- | --- | --- | --- | --- | --- | --- | --- | --- | --- | --- | --- | --- | --- | --- | --- | --- | --- | --- | --- | --- | --- | --- | --- | --- | --- | --- | --- | --- | --- | --- | --- | --- | --- | --- | --- | --- | --- | --- | --- | --- | --- | --- | --- | --- | --- | --- | --- | --- | --- | --- | --- | --- | --- | --- | --- | --- | --- | --- | --- | --- | --- | --- | --- | --- | --- | --- | --- | --- | --- | --- | --- | --- | --- | --- | --- | --- | --- | --- | --- | --- | --- | --- | --- | --- | --- | --- | --- | --- | --- | --- | --- | --- | --- | --- | --- | --- | --- | --- | --- | --- | --- | --- | --- | --- | --- | --- | --- | --- | --- | --- | --- | --- | --- | --- | --- | --- | --- | --- | --- | --- | --- | --- | --- | --- | --- | --- | --- | --- | --- | --- | --- | --- | --- | --- | --- | --- | --- | --- | --- | --- | --- | --- | --- | --- | --- | --- | --- | --- | --- | --- | --- | --- | --- | --- | --- | --- | --- | --- | --- | --- | --- | --- | --- | --- | --- | --- | --- | --- | --- | --- | --- | --- | --- | --- | --- | --- | --- | --- | --- | --- | --- | --- | --- | --- | --- | --- | --- | --- | --- | --- | --- | --- | --- | --- | --- | --- | --- | --- | --- | --- | --- | --- | --- | --- | --- | --- | --- | --- | --- | --- | --- | --- | --- | --- | --- | --- | --- | --- | --- | --- | --- | --- | --- | --- | --- | --- | --- | --- | --- | --- | --- | --- | --- | --- | --- | --- | --- | --- | --- | --- | --- | --- | --- | --- | --- | --- | --- | --- | --- | --- | --- | --- | --- | --- | --- | --- | --- | --- | --- | --- | --- | --- | --- | --- | --- | --- | --- | --- | --- | --- | --- | --- | --- | --- | --- | --- | --- | --- | --- | --- | --- | --- | --- | --- | --- | --- | --- | --- | --- | --- | --- | --- | --- | --- | --- | --- | --- | --- | --- | --- | --- | --- | --- | --- | --- | --- | --- | --- | --- | --- | --- | --- | --- | --- | --- | --- | --- | --- | --- | --- | --- | --- | --- | --- | --- | --- | --- | --- | --- | --- | --- | --- | --- | --- | --- | --- | --- | --- | --- | --- | --- | --- | --- | --- | --- | --- | --- | --- | --- | --- | --- | --- | --- | --- | --- | --- | --- | --- | --- | --- | --- | --- | --- | --- | --- | --- | --- | --- | --- | --- | --- | --- | --- | --- | --- | --- | --- | --- | --- | --- | --- | --- | --- | --- | --- | --- | --- | --- | --- | --- | --- | --- | --- | --- | --- | --- | --- | --- | --- | --- | --- | --- | --- | --- | --- | --- | --- | --- | --- | --- | --- | --- | --- | --- | --- | --- | --- | --- | --- | --- | --- | --- | --- | --- | --- | --- | --- | --- | --- | --- | --- | --- | --- | --- | --- | --- | --- | --- | --- | --- | --- | --- | --- | --- | --- | --- | --- | --- | --- | --- | --- | --- | --- | --- | --- | --- | --- | --- | --- | --- | --- | --- | --- | --- | --- | --- | --- | --- | --- | --- | --- | --- | --- | --- | --- | --- | --- | --- | --- | --- | --- | --- | --- | --- | --- | --- | --- | --- | --- | --- | --- | --- | --- | --- | --- | --- | --- | --- | --- | --- | --- | --- | --- | --- | --- | --- | --- | --- | --- | --- | --- | --- | --- | --- | --- | --- | --- | --- | --- | --- | --- | --- | --- | --- | --- | --- | --- | --- | --- | --- | --- | --- | --- | --- | --- | --- | --- | --- | --- | --- | --- | --- | --- | --- | --- | --- | --- | --- | --- | --- | --- | --- | --- | --- | --- | --- | --- | --- | --- | --- | --- | --- | --- | --- | --- | --- | --- | --- | --- | --- | --- | --- | --- | --- | --- | --- | --- | --- | --- | --- | --- | --- | --- | --- | --- | --- | --- | --- | --- | --- | --- | --- | --- | --- | --- | --- | --- | --- | --- | --- | --- | --- | --- | --- | --- | --- | --- | --- | --- | --- | --- | --- | --- | --- | --- | --- | --- | --- | --- | --- | --- | --- | --- | --- | --- | --- | --- | --- | --- | --- | --- | --- | --- | --- | --- | --- | --- | --- | --- | --- | --- | --- | --- | --- | --- | --- | --- | --- | --- | --- | --- | --- | --- | --- | --- | --- | --- | --- | --- | --- | --- | --- | --- | --- | --- | --- | --- | --- | --- | --- | --- | --- | --- | --- | --- | --- | --- | --- | --- | --- | --- | --- | --- | --- | --- | --- | --- | --- | --- | --- | --- | --- | --- | --- | --- | --- | --- | --- | --- | --- | --- | --- | --- | --- | --- | --- | --- | --- | --- | --- | --- | --- | --- | --- | --- | --- | --- | --- | --- | --- | --- | --- | --- | --- | --- | --- | --- | --- | --- | --- | --- | --- | --- | --- | --- | --- | --- | --- | --- | --- | --- | --- | --- | --- | --- | --- | --- | --- | --- | --- | --- | --- | --- | --- | --- | --- | --- | --- | --- | --- | --- | --- | --- | --- | --- | --- | --- | --- | --- | --- | --- | --- | --- | --- | --- | --- | --- | --- | --- | --- | --- | --- | --- | --- | --- | --- | --- | --- | --- | --- | --- | --- | --- | --- | --- | --- | --- | --- | --- | --- | --- | --- | --- | --- | --- | --- | --- | --- | --- | --- | --- | --- | --- | --- | --- | --- | --- | --- | --- | --- | --- | --- | --- | --- | --- | --- | --- | --- | --- | --- | --- | --- | --- | --- | --- | --- | --- | --- | --- | --- | --- | --- | --- | --- | --- | --- | --- | --- | --- | --- | --- | --- | --- | --- | --- | --- | --- | --- | --- | --- | --- | --- | --- | --- | --- | --- | --- | --- | --- | --- | --- | --- | --- | --- | --- | --- | --- | --- | --- | --- | --- | --- | --- | --- | --- | --- | --- | --- | --- | --- | --- | --- | --- | --- | --- | --- | --- | --- | --- | --- | --- | --- | --- | --- | --- | --- | --- | --- | --- | --- | --- | --- | --- | --- | --- | --- | --- | --- | --- | --- | --- | --- | --- | --- | --- | --- | --- | --- | --- | --- | --- | --- | --- | --- | --- | --- | --- | --- | --- | --- | --- | --- | --- | --- | --- | --- | --- | --- | --- | --- | --- | --- | --- | --- | --- | --- | --- | --- | --- | --- | --- | --- | --- | --- | --- | --- | --- | --- | --- | --- | --- | --- | --- | --- | --- | --- | --- | --- | --- | --- | --- | --- | --- | --- | --- | --- | --- | --- | --- | --- | --- | --- | --- | --- | --- | --- | --- | --- | --- | --- | --- | --- | --- | --- | --- | --- | --- | --- | --- | --- | --- | --- | --- | --- | --- | --- | --- | --- | --- | --- | --- | --- | --- | --- | --- | --- | --- | --- | --- | --- | --- | --- | --- | --- | --- | --- | --- | --- | --- | --- | --- | --- | --- | --- | --- | --- | --- | --- | --- | --- | --- | --- | --- | --- | --- | --- | --- | --- | --- | --- | --- | --- | --- | --- | --- | --- | --- | --- | --- | --- | --- | --- | --- | --- | --- | --- | --- | --- | --- | --- | --- | --- | --- | --- | --- | --- | --- | --- | --- | --- | --- | --- | --- | --- | --- | --- | --- | --- | --- | --- | --- | --- | --- | --- | --- | --- | --- | --- | --- | --- | --- | --- | --- | --- | --- | --- | --- | --- | --- | --- | --- | --- | --- | --- | --- | --- | --- | --- | --- | --- | --- | --- | --- | --- | --- | --- | --- | --- | --- | --- | --- | --- | --- | --- | --- | --- | --- | --- | --- | --- | --- | --- | --- | --- | --- | --- | --- | --- | --- | --- | --- | --- | --- | --- | --- | --- | --- | --- | --- | --- | --- | --- | --- | --- | --- | --- | --- | --- | --- | --- | --- | --- | --- | --- | --- | --- | --- | --- | --- | --- |
|  |  |  |  |
|  |  |  |  |
|  |  |  |  |
|  |  |  |  |
|  |  |  |  |
|  |  |  |  |
|  |  |  |  |
|  |  |  |  |
|  |  |  |  |
|  |  |  |  |
|  |  |  |  |
|  |  |  |  |
|  |  |  |  |
|  |  |  |  |
|  |  |  |  |
|  |  |  |  |
|  |  |  |  |
|  |  |  |  |
|  |  |  |  |
|  |  |  |  |
|  |  |  |  |
|  |  |  |  |
|  |  |  |  |
|  |  |  |  |
|  |  |  |  |
|  |  |  |  |
|  |  |  |  |
|  |  |  |  |
|  |  |  |  |
|  |  |  |  |
|  |  |  |  |
|  |  |  |  |
|  |  |  |  |
|  |  |  |  |
|  |  |  |  |
|  |  |  |  |
|  |  |  |  |
|  |  |  |  |
|  |  |  |  |
|  |  |  |  |
|  |  |  |  |
|  |  |  |  |
|  |  |  |  |
|  |  |  |  |
|  |  |  |  |
|  |  |  |  |
|  |  |  |  |
|  |  |  |  |
|  |  |  |  |
|  |  |  |  |
|  |  |  |  |
|  |  |  |  |
|  |  |  |  |
|  |  |  |  |
|  |  |  |  |
|  |  |  |  |
|  |  |  |  |
|  |  |  |  |
|  |  |  |  |
|  |  |  |  |
|  |  |  |  |
|  |  |  |  |
|  |  |  |  |
|  |  |  |  |
|  |  |  |  |
|  |  |  |  |
